# Rhizosphere Microbiomes of Citrus Plants in Historically Undisturbed 100-Year-Old Groves Appear to Mitigate Susceptibility to Citrus Greening Disease

**DOI:** 10.3390/microorganisms13040763

**Published:** 2025-03-27

**Authors:** Nwadiuto Esiobu, Karim Dawkins, Yasmine Sanhaji, Melissa Voorn, Karina Murillo, Zachary Hill, Faiza Naeem, Joel Edouard, Donald McCorquodale

**Affiliations:** 1 Microbial Biotechnology Laboratory, Biology Department, Florida Atlantic University, 777 Glades Road, Boca Raton, FL 33431, USA; dawkinsk@fau.edu (K.D.); yasmine.sanhaji@fau.edu (Y.S.); mvoorn@fau.edu (M.V.); kmurillo2015@fau.edu (K.M.); zhill2020@fau.edu (Z.H.); fnaeem2019@fau.edu (F.N.); edouardj2021@fau.edu (J.E.); 2 Department of Marine and Environmental Sciences, Nova Southeastern University, 8000 N Ocean Dr, Dania Beach, FL 33004, USA; mccorquodale@nova.edu

**Keywords:** agrochemical usage, beneficial bacteria of citrus, citrus greening disease, microbiome, plant health, soil health, undisturbed citrus groves, Verrucomicrobia

## Abstract

Microbiome studies aimed at combating the citrus greening devastation caused by *Liberibacter asiaticus* abound. However, the role of farming practices, such as the massive use of herbicides, pesticides, and inorganic fertilizers on specific taxa and plant population immunity remains an important inquiry. To test our hypothesis that agricultural practices in managed Citrus groves induce root microbiome dysbiosis, potentially rendering citrus readily susceptible to citrus greening disease (CGD), we compared the CGD and root microbiome status of citrus plants in a rare > 130-year-old grove (no anthropogenic influence) to those of managed Valencia groves (symptomatic and asymptomatic). Citrus greening disease was detected by qPCR using the HLBa/HLBs/HLBp primer/probe combination, while root microbiome community structure was determined using 16S rDNA amplicon sequencing. The prevalence of CGD among citrus growing in the undisturbed, healthy soils was zero (Ct values > 36), while symptomatic and asymptomatic Valencia from managed groves was 100% positive (Ct < 34). Known beneficial plant symbionts (Actinomycetales, *Bradyrhizobium*, Verrucomicrobia, etc.) from Phylum Actinobacteria and Proteobacteria were depleted in the rhizosphere of the managed sites. This dysbiotic shift was characterized by enrichment with *Acidobacterium*, *Nitrospira,* and *Sphingomonas* spp. In highly infected Valencia oranges, beneficial taxa of the Alphaproteobacteria declined significantly (from 20–25% to 10–15%), while *Bacillus* sp. (a Firmicutes) was enriched 13-fold. Simpson and Shannon diversity indices were similar for all plant microbiomes except the heavily infected Valencia, which exhibited low diversity (*p* < 0.05), indicating that diversity indices alone are not reliable measures of soil health or rhizobiome homeostasis. Large reservoirs of known and novel putative beneficial rhizosphere microbes in undisturbed sites supported zero CGD, despite proximity to the managed sites where diverse non-beneficial taxa coincided with high disease rates. Supplementing the use of agrochemicals with carefully designed microbial products for plant disease control and sustainable soil health deserves acute attention.

## 1. Introduction

Citrus greening, otherwise known as Huanglongbing (HLB), is one of the most devastating citrus diseases in the world. First discovered in 1920, the disease migrated from Asia to Florida in 2005 and subsequently to other sub-tropical US states, including Texas, Louisiana, and California. It is caused by a difficult-to-culture Gram-negative endophytic bacterium of the *Alphaproteobacteria* class called *Candidatum* Liberibacter asiaticus, which is transmitted by a Sternorrhynchian insect vector—the Asian citrus psyllid, *Diaphorina citri* [1]. The Asian citrus psyllid is the most heat-tolerant of all three types, which also includes the Americanus and Africanus citrus psyllids, but the Asian type has been found extensively in Florida [1]. Early symptoms of its infection on leaves include yellowing of the veins and adjacent tissues, followed by asymmetrical chlorosis—symptoms that resemble zinc and other mineral deficiencies. The disease leads to premature defoliation, dieback of twigs, decay of feeder rootlets and lateral roots, decline in vigor, and could ultimately lead to the death of the entire plant. Most affected trees have stunted growth, bear multiple off-season flowers (most of which fall off), and produce small, irregularly shaped fruit with a thick, pale peel that remains green at the bottom and tastes very bitter [1,2]. Citrus greening disease (CGD) is prevalent in 80% of groves within the citrus industry in South Florida. Annual losses between 30 and 100% have decimated the 10.8 billion-dollar industry. Since its introduction in 2005 in Miami-Dade, Florida, it has rapidly spread to more than 37 counties and nine other states. The rapid spread was possibly fueled by dysbiosis within the rhizosphere stemming from agricultural management practices that rely on the heavy use of agrochemicals among other factors.

Research on citrus root microbiomes to combat the citrus greening devastation abounds. The use of microbiome-based solutions has produced mixed outcomes. Optimizing these interventions requires an understanding of the functional and taxonomic niches of core plant microbiomes. Understanding why the newly introduced Ca. L. asiaticus pathogen swept rapidly through most Florida Citrus groves is important for proposing solutions for future outbreaks. Knowledge about plant population immunity and the roles played by microbiomes is rapidly accumulating. The translational interpretations and applications of these study outcomes in specific practical situations are critical for sustainable agriculture.

It has long been known that HLB infection causes phloem disruption, sucrose accumulation, and plugging of the sieve pores linked to callose deposition and not HLB aggregates [3]. The molecular basis of the disease is also well understood. Many plant genes are either upregulated (pathogenesis/stress response, phytohormones, sugar and protein metabolism, and several unknown genes) or downregulated (cell cycle, cell wall and lipid metabolism, and protein kinases) during infection, suggesting an intense perturbation of overall plant metabolism [4]. Genes, such as the plant defense and disease-resistance response (PR) genes, may provoke a sort of “plant hypersensitive” or “chronic inflammation” damage, which is reflected in callose deposition in and around the phloem. Of greater note, perhaps, is that about 50% of PR genes were downregulated in sweet oranges by Ca. *Libericater sp* infection [5], while inoculating citrus plants with beneficial Burkholderia isolated from healthy citrus plants enhanced the expression of PR genes and salicylic acid-mediated resistance, demonstrating the high potential of biocontrol agents in controlling HLB infection. Further evidence comes from the observation that rhizospheric non-virulent *P. syringae* induced the expression of the non-host resistance gene—NHO1 in *Arabidopsis sp*., whereas the virulent strains of the same bacterium (Pst DC3000) were able to cause disease by suppressing the expression of the photo-immunity (NH01 plant defense mechanism) via a jasmonic acid (JA) signaling pathway [6]. Other microorganisms reported to confer pathogen suppression in soils include bacteria (members of the Proteobacteria, Verrucomicrobia, and Firmicutes) and fungi—Ascomycota or *M. circinelloides*, *P. oxalicum*, *A. niger,* and *A. flavus* [7,8,9].

Recent advances in disease progression implicate specific microorganisms associated with disease-induced dysbiosis and a microbe-mediated immune response model [10]. There was a transition from beneficial microorganisms such as *Glomeromycota*, *Camptohophora* sp., and *Methylobacterium* sp. to more competitive and pathogenic species such as *Fusarium* sp., *Giberella* sp., *Streptomyces* sp. during heavy infection, and finally, to saprophytic species as the disease progressed to the later stages when the trees had almost died [11]. Root exudates also provide nourishment for specific microbes, supporting plant immunity through inhibiting the growth of invasive pathogens [12].

Current control methods for CGD involve the removal of psyllid vectors using insecticides [13], chemotherapy treatments using antibiotics that target the HLB pathogen [14], and the disinfection and immediate removal of infected plants. In larger and more commercial citrus groves, these control methods have been insufficient in stemming the spread of the disease [13]. Genetic transformation of the citrus plant, the HLB pathogen, and the psyllid vector provide high expectations for the development of disease-resistant cultivars and/or amelioration of the bacterial pathogen and insect vector control [13]. The use of a biocontrol agent—*Tamarixia radiata*—has been explored in Texas, also showing great potential, where up to 90% of psyllids were reduced post-treatment [15]. Genomic solutions are being explored, so solving the growing HLB challenge will entail a concerted multidisciplinary effort.

The plant microbiome, or phytobiome, plays an integral role in the overall health and disease suppression [8,16] of different agricultural and non-agricultural plants and is highly sensitive to changes in the environment and varying stress conditions [17]. The rhizosphere, which consists of soil approximately 1 mm from the plant root surface, is one of the most diverse and enriched habitats for microbial life and consists of many beneficial and antagonistic plant-associated micro-organisms. The pivotal role played by these root microbiomes in plant survival and competition is emerging [17], and the manipulation of the rhizobiome could have important implications for vegetation health and cover [18]. Numerous studies using amplicon, metagenomic, and meta-transcriptomic sequencing analyses have assessed the effects of different environmental conditions and diseased states on the microbial community structure of the rhizosphere of a vast array of plants, including invasive plants, Arabidopsis, rice, soybean, and citrus [8,19,20,21]. As a part of the mandate of the International Citrus Microbiome Consortium, it is crucial to determine the effect of different citrus management strategies (herbicide, fertilizer treatment) on the citrus microbiome and develop methods to engineer the microbiome for sustainable plant production. Recent research has shown a consistent and core microbiome present in citrus varieties across the globe and may provide insights into how these microbiomes can be employed to increase sustainable crop production [20].

In this study, we leveraged a very rare opportunity to access undisturbed citrus groves of the 1880s to capture potentially unknown microbiomes of citrus. The second goal was to evaluate the impact of routine citrus cultivation practices (which include copious application of agrochemicals) on root microbiomes by contrasting microbial communities on managed groves in the same vicinity with those of the undisturbed terrain. We then compared the profiles of 16S rDNA amplicon sequence variants (ASVs) with the prevalence of greening disease in the plants to infer the effect of dysbiosis on susceptibility to pathogens like *Liberibacter asiaticus*. Finally, we mined the data from the Global Citrus Consortium to determine the occurrence of published core citrus microbiomes in our study sites. While it might be too late to reverse the damage and rescue the Florida Citrus industry, data from this study would inform policy on soil health and sustainable management practices for other important agricultural crops like sugarcane. To the best of our knowledge, this is one of the first studies of rhizosphere microbiomes of historically undisturbed century-old citrus groves, free of anthropogenic impacts.

## 2. Materials and Methods

### 2.1. Site Description

Two varieties of sweet orange (*Citrus sinensis*)—Valencia and pineapple orange—along with sour orange (*Citrus aurantium*), grapefruit (*Citrus paradisi*), and sour lemon (*Citrus limon*) were assessed visually and at the molecular level for *Candidatus* L *asiaticus* infection throughout different groves in Central Florida. East and West Valencia oranges were located in the Hendry County grove (Site 1—26.6105° N, 81.0755° W) and were found to have visual foliar signs of Huanglongbing infection, with the West Valencia oranges showing greater signs of infection than East Valencia. Both East and West Valencia oranges were positive for Ca. L *asiaticus*. These groves had been cultivated for over 30 years with consistent herbicide and other chemical use (pers comm. McCoquerdale). Herbicide and fertilizer treatments involved the use of glyphosate (Roundupâ) applied at a rate of 0.125–0.37 lbs/acre and a commercial citrus fertilizer (6-4-6 NPK ratio) at an application rate of 120–200 lbs/acre.

**Figure 1:**
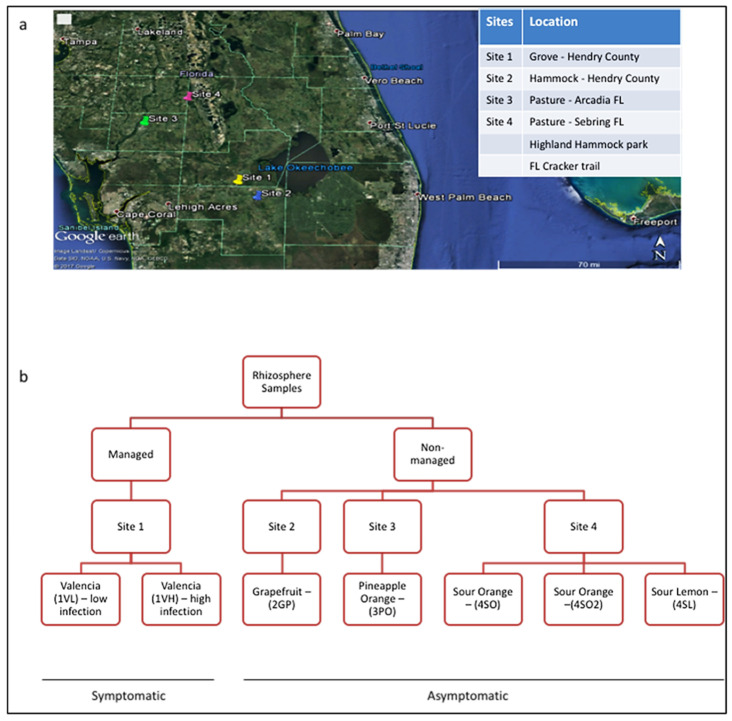
(**a**) Map of Central Florida showing the four different sampling sites, where rhizosphere soil was collected from each citrus species and variety. (**b**) Sample design layout showing the location of each citrus species/variety and the presence or absence of citrus greening symptoms.

Natural stands of grapefruit were located on Site 2, a historical, isolated, and non-cultivated hammock area in Hendry County, with fruits that had no visual symptoms of Ca. L *asiaticus* infection. Site 3 was another isolated area in Arcadia, FL (27.2159° N, 81.8584° W), lacking anthropogenic influences with a grove of Greenmount pineapple oranges. On site 4, in Sebring, FL, wild-grown sour orange and sour lemon were found in Highland Hammock State Park and Florida Cracker Trail. These plants exhibited no signs of infection. A sample map of the sampling sites and their respective levels of infection is shown in Figure 1. Root samples from different plant types were collected using the grab method, in duplicate, and sent immediately to the lab in a cooler with ice between November 2016 and February 2017. The roots were shaken to remove loosely adhering soil, while the rhizosphere soil was scraped off using forceps and used for the study.

### 2.2. DNA Extraction and Validation

Two grams of total rhizosphere soil sample, instead of 0.75 g (recommended by the manufacturer), were collected from the rhizosphere of each plant along with bulk soil and extracted using the MoBio Power Soil Kit (Mo Bio Laboratories Inc., Carlsbad, CA, USA). This modified in situ technique significantly increased taxa representation and DNA yield from the sample matrix. The two grams of soil samples were vortexed in 10 mL sterile 1X phosphate buffer for 10 min to dislodge bacterial cells, which were then harvested into a pellet at 13,000 rpm for 5 min and subjected to the MoBio Power Soil Kit according to the manufacturer’s instructions.

The DNA concentration and purity of each sample were measured using the Nanodrop 2000c spectrophotometer (Thermo-Fisher Scientific, Waltham, MA, USA). Extracted samples were also run on a 1% agarose gel (1X TAE) at 90 V for 45 min to visually verify the purity of the samples by determining whether DNA bands were intact or degraded. To further confirm the integrity of the extracted DNA, the bacterial 16S rDNA was amplified using the universal primers 1492 Reverse sequences (5′GGTTACCTTGTTACGACTT-3′) and 27 Forward (5′-AGAGTTTGATCCTGGCTCAG-3′) that target the V1-V9 region of the 16S rDNA gene and produce an approximate 1500 base pairs (bp) fragment. Reaction mixtures were incubated for 4 min at 94 °C for denaturation, followed by 35 cycles consisting of 1 min at 94 °C, annealing for 30 s at 45 °C, and extension for 2 min at 72 °C using Taq Polymerase from the 2X Promega PCR master mix (Promega Corp., Madison, WI, USA), along with 0.4 µM of each primer, 10 µg bovine serum albumin (Promega Corp., Madison, WI, USA), 2 mM MgCl_2,_ and approximately 20 ng of DNA template for each sample in a final 25 µL reaction mix. Four (4 µL) microliters of PCR products were run using gel electrophoresis on a 1% agarose (*w*/*v*) gel at 90 V for 45 min to view the expected ~1500 bp band size.

### 2.3. Ca. L asiaticus Detection

Leaf samples were collected from each plant and extracted using the Wizard Magnetic 96 DNA Plant System Instrument (Promega Corp., Madison, WI, USA) according to the manufacturer’s conditions. The qPCR reactions were performed at the University of Florida SWFREC-HLB Laboratory in Immokalee, FL, using the primer/probe set: HLBas (CGAGCGCGTATGCAATACG)/HLBr (CTACCTTTTTCTACGGGATAACGC)/HLBp (AGACGGGTGAGTAACGCG), which amplifies a section of the Las gene on the Applied Biosystems 7500 Fast Real-Time PCR (Applied Biosystems, Foster City, CA, USA) and targets the 16S rDNA. Briefly, qPCR reactions were performed in triplicate in a 25 μL reaction using 2× Quantitect Probe PCR master mix (Qiagen, Valencia, CA, USA), 0.8 μM of each primer, 0.4 μM of probe (IDT, Coralville, IA, USA), and an appropriate amount of template DNA. The PCR conditions were 50 °C for 2 min, 95 °C for 15 min, 45 cycles of each 94 °C for 15 sec, and 60 °C for 1 min. Samples positive for the presence of HLB, resulted in Ct values less than 36, while samples negative for the presence of HLB, resulted in Ct values > or = to 36 (Table 1). The threshold was automatically assigned by the analysis software. Lower Ct values indicate higher initial template conc in the well. If sample DNA contains *Las* DNA, an amplicon will be created in the PCR reaction, and the amount will be reflected in the Ct value. A Ct of 36 or less was considered positive for detection. The range between 32 and 36 Ct was considered putatively positive, and trees tested in this range normally resulted in lower Ct values in subsequent assays [13].

### 2.4. Processing of Sequence Data Using QIIME and CosmosID Platform

The forward and reverse reads were paired using the join_paired_ends.py script in QIIME (Quantitative Insights in Microbial Ecology) [22]. Joined paired-end reads were then quality-filtered using the q30 standard, where the probability of an incorrect base call is 99.9%. Sequences that did not meet these criteria were removed. A second quality control step—chimera check (identify_chimeric_seqs.py)—was performed to identify and remove chimeric sequences. The operational taxonomic unit (OTU) table in ‘biome’ format was then generated by picking OTUs that were aligned against the Greengenes database [23] for 16S rDNA and also unaligned sequence OTUs using open reference OTU picking at the 97% cut-off. Additional analysis of the OTU tables, using alpha and beta diversity measures along with a taxonomy summary indicating the relative abundances for each sample, was performed using bubble charts and heatmaps from CosmosID. Alpha and Beta analyses were generated by using the QIIME and R software version 4.1.0. Both analyses were rarefied by using the median eigenvalues from the number of reads obtained for all samples. Core OTUs were computed using QIIME version 1.9.1 with the script ‘compute_core_microbiome.py’. The relative abundance of the different phyla observed was calculated from the total number of core OTUs for the rhizosphere of citrus plants from the managed and non-managed sites. The original 16S sequence files were submitted to NCBI with project accession #PRJNA680160 and sample accession numbers SRX9584730-43.

### 2.5. Statistical Analysis

The statistical significance of the OTUs, Shannon’s diversity index, and observed relative abundance and diversity measures was tested using two-way ANOVAs at the 95% confidence limit, followed by the post hoc Tukey HSD test if significance was found.

## 3. Results

### 3.1. Detection of Ca. L. asiaticus in the Rhizosphere Samples

The qPCR analysis used the primer/probe set HLBas/HLBr/HLBp, which targeted the 16S rDNA of Ca. L. *asiaticus,* helping to confirm the presence of the pathogen in leaf samples of the different plant types: Valencia, pineapple orange, grapefruit, sour lemon, and sour orange (Table 1). A Ct value of 36 was used as the cut-off point for positive detection, where samples with Ct values below 36 were deemed positive for the presence of the HLB pathogen. Table 1, which shows the qPCR results for each plant type, revealed the presence of HLB in Valencia plants in a currently managed grove. The grapefruit, pineapple orange, sour orange, and sour lemon plants were devoid of the HLB pathogen, with Ct values of 36 or higher for each triplicate.

Strikingly, all samples from managed groves tested positive for *L. asiaticus*, even when asymptomatic. No plant from unmanaged sites, devoid of anthropogenic effects, tested positive for the disease.

### 3.2. Reads of 16S rDNA Amplicon and Operational Taxonomic Units (OTUs)

The bacterial community structure of the rhizosphere of infected East and West Valencia citrus plants (*Citrus sinensis*) was evaluated at site 1 using the total genomic DNA from the rhizosphere (n = 4). Similarly, the bacterial community structure of a pineapple orange variety (*Citrus* × *sinensis*) (n = 2), grapefruit (*Citrus* × *paradisi*) (n = 2), sour orange (*Citrus* × *aurantium*) at two different sites (n = 4), and sour lemon (*Citrus limon*) (n = 2) all located on non-cultivated sites, were also evaluated. A total of 195,122 sequenced reads was obtained after the initial quality filtering at Q30 (99.9% base call accuracy). After normalization, the sour lemon rhizosphere from a non-cultivated site had the highest average number of reads (15,925), accounting for 16% of total reads, while the highly infected Valencia orange had the lowest average number of reads (11,705), representing 12% of total reads. The Valencia oranges with ‘low infection’ averaged the second-highest number of reads (15,130). The sour orange at both managed sites reported 12,450 and 14,946 reads, while the grapefruit and infected pineapple orange variety had 14,325 and 13,079 reads, respectively. A one-way ANOVA of bacterial reads revealed no significant difference between plant types, infected or non-infected (*p* = 0.77), and no significant difference in means was found between East and West Valencia oranges using an unpaired T-test (*p* = 0.49). Bacterial operational taxonomic units (OTUs) were generated using an open reference allocation, where sequences with >97% similarity were grouped as a single OTU using the UCLUST algorithm. The total number of OTUs obtained (12,346) for all the samples is shown in Table 1.

**Figure 2:**
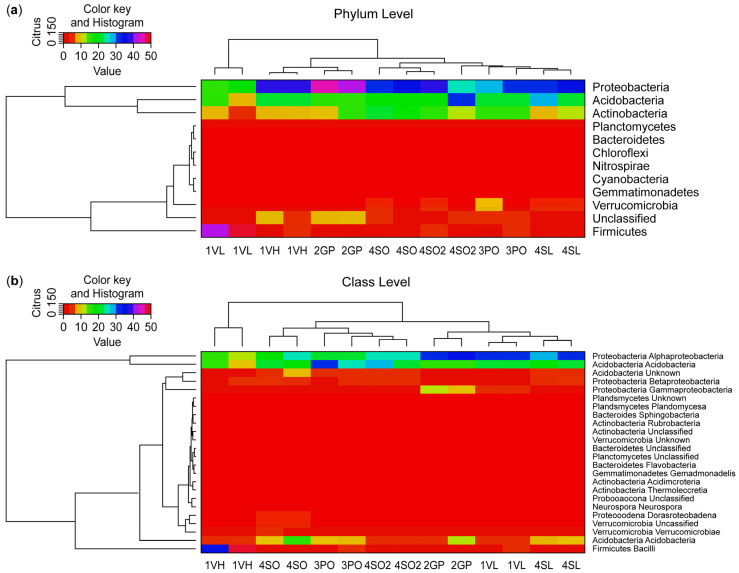
Heatmap showing (**a**) the relative abundance of 12 top 16S bacterial phyla seen under the rhizosphere of the different citrus plant types and (**b**) the relative abundance of the 24 top 16S bacterial classes seen under the rhizosphere of the different citrus plant types.

**Figure 3:**
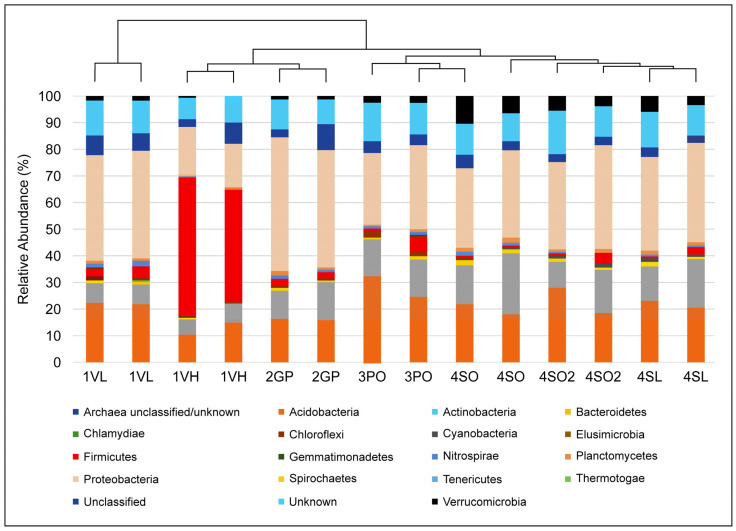
Relative abundance of bacteria phyla under managed and unmanaged citrus groves of the citrus varieties. At the phylum level, there is an insignificant difference between managed and historical groves even though striking variances emerge at lower taxonomic taxa. Noteworthy is the dysbiotic shift that replaced Proteobacteria immune-supporting bacteria and other phyla with Bacillus Firmicutes in the heavily infected Valencia (IVH) citrus rhizosphere under managed groves.

A total of seventeen known and three unclassified bacteria phyla were identified in this study. Heat maps and tree diagrams are provided in Figure 2, showing (a) the relative abundance of 12 top 16S bacterial phyla seen under the rhizosphere of the different Citrus plant types and (b) the relative abundance of the 24 top 16S bacterial classes seen under the rhizosphere of the different Citrus plant types. Percentages of total phylum reads by sample site are shown in Figure 3. The top phylum observed in the rhizosphere of all sample plant types was the Proteobacteria, which amounted to 63,791 reads (32.7% of total sequences obtained). By contrast, in the West Valencia rhizosphere (highly symptomatic), the phylum *Firmicutes* was the most prevalent, accounting for 74% of its total reads. The *Acidobacteria* and *Actinobacteria* registered 39,192 (20%) and 23,525 (12%) reads, respectively, in all plant types. *Firmicutes* and *Verrucomicrobia* rounded out the top five with 15,295 (7.8%) and 6218 (3.2%) reads, respectively, in all plant types.

Similarly, the sour lemon rhizosphere had the highest average number of OTUs (1029), while the infected West Valencia plant rhizosphere had the lowest average number of OTUs (476). The East Valencia rhizosphere had 1000 OTUs, while the grapefruit and pineapple orange had 779 and 868 OTUs, respectively. The two sour orange plants at Site 4 had 1033 and 986 OTUs, respectively, in the rhizosphere. A two-way ANOVA revealed a significant difference, diminishing chances for random occurrence between the OTUs of the different plant types and sites (*p* = 0.0062), where Valencia orange (1VH) was significantly different from Valencia (1VL), sour orange (4SO/4SO2), and sour lemon (4SL), using the Tukey HSD post hoc test at the 95% confidence level.

### 3.3. Bacterial Community Structure of the Rhizosphere of the Low-Level Infection Versus No Infection in Non-Managed Citrus Varieties

**Figure 4:**
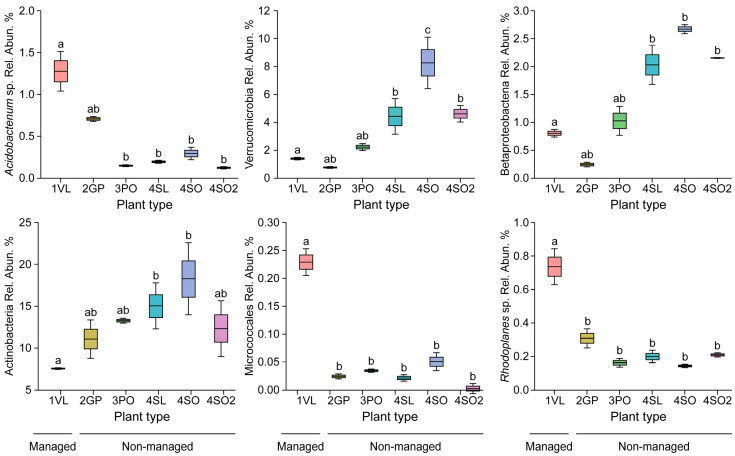
The relative abundance of key bacterial taxa of the core OTUs recovered from the rhizosphere of the managed and non-managed citrus varieties reveals a remarkable consistency and homeostasis of all non-managed undisturbed sites, in sharp contrast to managed groves. Numbers with same letter are not significantly different.

The rhizosphere of the managed Valencia site with low infection had minimal changes in the rhizosphere due to HLB infection, compared with samples from the non-managed historical sites 2, 3, and 4, based on diversity indices at the phylum level. The total number of core OTUs calculated at the 95% level (the fraction of samples required for an OTU to be considered a ‘core’) was 193 for the non-managed sites. Eleven (11) phyla were part of the core OTUs in the non-managed sites, along with different unknown/unclassified phyla, while 12 phyla were observed in the managed site. Of the known phyla, *Proteobacteria*, *Acidobacteria*, and *Actinobacteria* exhibited the highest relative abundance represented in the core OTUs for both managed and non-managed sites. These three major phyla had a lower relative abundance in the rhizosphere of the managed site than the other non-managed sites. There was a small reduction in the relative abundance of Proteobacteria at the Valencia-managed site due to the depletion of taxa from the *Alphaproteobacteria*, including *Bradyrhizobium* sp., *Betaproteobacteria,* and *Actinomycetales,* as displayed in Table 2 and Figure 4. There was, however, a two-fold increase in the relative abundance of *Bacteriodetes* and Firmicutes, including *Sphingomonas* sp. and *Bacillus* sp., in the managed site rhizosphere compared to the non-managed sites.

**Figure 5:**
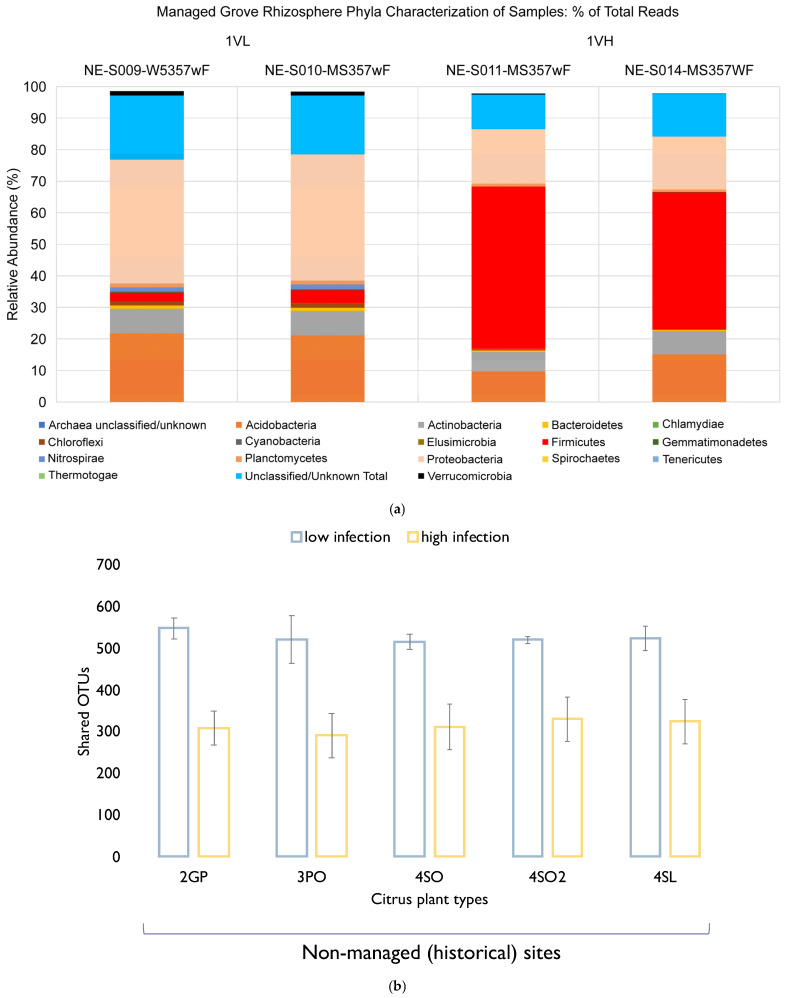
(**a**) Rhizosphere bacterial community structure of Valencia with high- and low-level infection in the managed grove. (**b**) Number of shared citrus rhizosphere bacterial OTUs between the non-managed historical sites and the managed sites (low + high infection): 2GP—Site 2 grapefruit, 3PO—Site 3 pineapple orange, 4SO—Site 4 sour orange, 4SO2—Site 4 sour orange, 4SL—Site 4 sour lemon.

The grapefruit, pineapple orange, sour orange, and sour lemon plants were located on non-managed sites devoid of anthropogenic involvement. The top five phyla throughout the different plant types were the same for the East Valencia plants and included Proteobacteria as the most prevalent, with the grapefruit rhizosphere showing the highest relative abundance (43.1%), followed by sour lemon (34.8%), sour orange at Hart Cracker Trail (HCT) (34.3%), sour orange at Hart Highway (29.4%), and pineapple orange (28.5%) (Figure 5a). The *Alphaproteobacteria* was the most prevalent class, representing >60% of Proteobacteria reads across the plant types. The *Rhizobiales* order was the most prevalent, with >60% of *Alphaproteobacteria* reads throughout the different plant types. The *Alphaproteobacteria* class in these non-managed sites had a higher average relative abundance (25–35%) compared to the highly infected West Valencia (10–15%), as shown in Figure 5b. The number of reads for members of the *Rhizobiales* order was enhanced under the non-managed citrus plants, with two- to three-fold increases in the number of reads for *Bradyrhizobium* sp., *Rhodoplanes* sp., and *Rhizobium* sp. The prevalence of the *Firmicutes* phylum was substantially reduced (~13 fold) under the rhizosphere of the non-managed citrus plants (0.48–3.3%) (Figure 5a) and was significantly different. The phylum was dominated by Bacillus sp. from the class Bacilli. The relative abundance of the *Actinobacteria* phylum was elevated under the rhizosphere of the non-managed citrus plants (11.1–18.3%), which contained many unclassified Actinobacteria classes and *Actinomycetales* orders. The *Kribella* sp. and an unclassified *Norcadiodaceae* family were present in the non-managed citrus plants but absent from the rhizosphere of the symptomatic Valencia (1VL) plant at the managed site. The *Verrucomicrobia* phylum showed a five-fold increase in relative abundance under the non-managed citrus plants (2.2–8.2%), except in the grapefruit rhizosphere (0.75%) (Figure 5a).

### 3.4. Rhizosphere Bacterial Community Structure of Valencia with High and Low Levels of Infection Versus the Managed Grove

The Valencia orange samples were both positive for the presence of the HLB pathogen, but one section of the grove had a high level of infection where the symptoms on the plants were widespread. The other Valencia plants sampled had a low level of infection or milder symptoms. There were pronounced differences between the rhizospheres of the Valencia oranges, even though both were infected. The bacterial community structure of the mildly infected Valencia rhizosphere more closely mimicked that of the non-managed citrus plants’ rhizospheres. Of the 17 known phyla found in the different plant types, only 12 were observed in the Valencia (1VH) rhizosphere, while 14/17 known phyla were found under the Valencia (1VL) rhizosphere. The top five known phyla and their relative abundance under the Valencia (1VH) rhizosphere included Firmicutes (47.2%), *Proteobacteria* (16%), Acidobacteria (12.2%), Actinobacteria (6.7%), and *Verrucomicrobia* (0.25%). The top five known phyla under the Valencia (1VL) rhizosphere were Proteobacteria (37.4%), *Acidobacteria* (21.5%), *Actinobacteria* (7.6%), *Firmicutes* (3.5%), and *Verrucomicrobia* (1.4%). The most striking bacterial community structure difference between the two Valencia orange rhizopheres was the prevalence of the Firmicutes (47.2%), where the relative abundance was more than thirteen-fold that of the low-symptomatic Valencia-1VL (3.5%)—(Figure 5).

Further examination indicates that the undisturbed citrus rhizosphere sites have a near 2-fold more shared OTUs with the low-symptomatic managed citrus rhizosphere compared with the highly symptomatic managed citrus rhizosphere (Figure 5b).

### 3.5. Alpha and Beta Diversity and Cluster Analysis of Samples in the Managed vs. Non-Managed Sites

**Figure 6:**
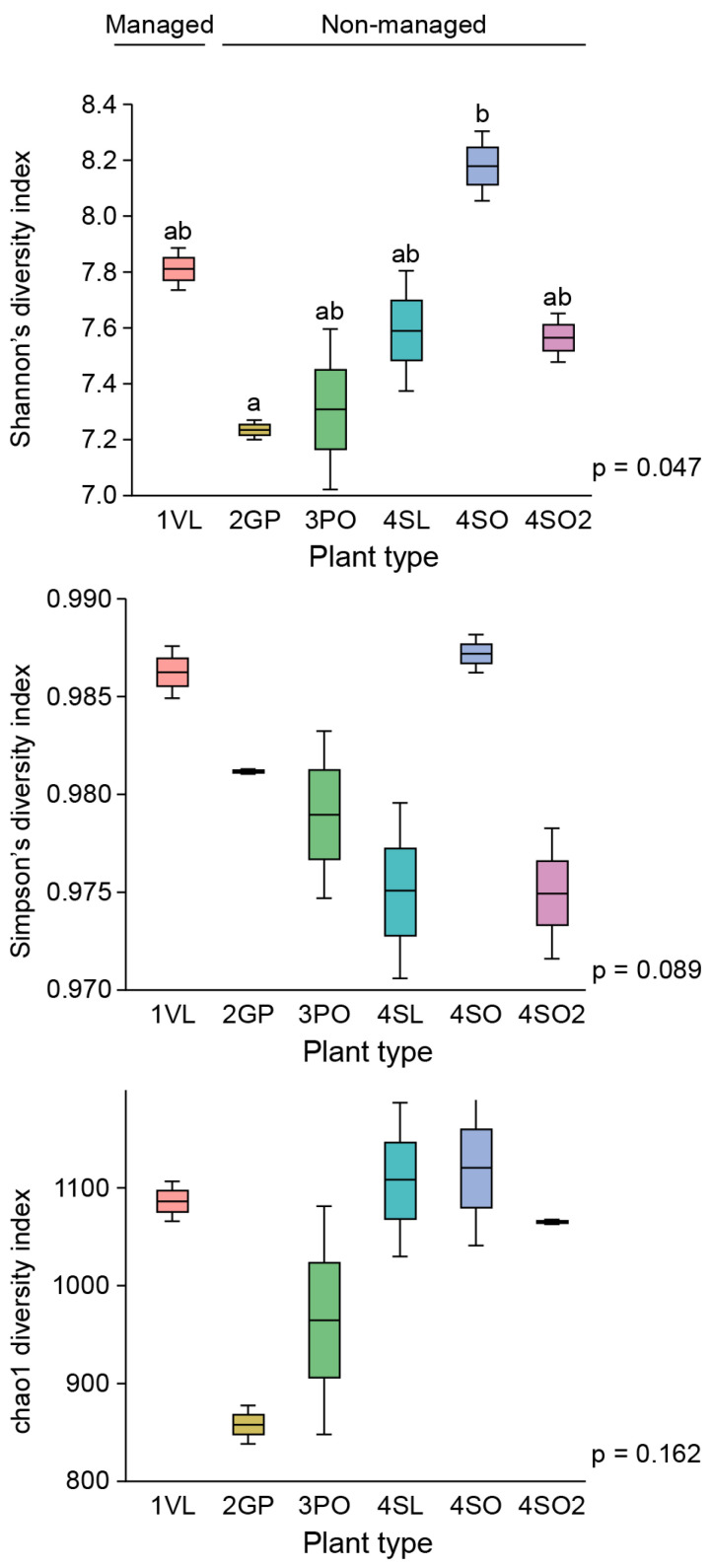
OTU alpha diversity (Shannon’s, Simpson’s, and chao1 index) of the different Citrus plant species/varieties in the managed vs. non-managed plant types and sites. 1VL—Site 1—Valencia (low symptomatic level), 2GP—Site 2 grapefruit, 3PO—Site 3 pineapple orange, 4SL—Site 4 sour lemon, 4SO—Site 4 sour orange, 4SO2—Site 4 sour orange. Items with the same letters are not significantly different.

Shannon’s diversity measure considers the relative abundance and evenness of the species present, while Simpson’s diversity index takes into account the number of species present, as well as the abundance of more dominant taxa. The Chao1 diversity index, on the other hand, measures the abundance of rarer taxa. All are representative of the alpha diversity within the samples. The average Shannon’s diversity index for the different plant types ranged from 5.5 to 8.15 (Figure 6). There was a significant difference between Shannon’s diversity in the grapefruit (2GP) and sour orange rhizosphere (4SO), with a weak *p*-value of 0.0047 confirmed by the Tukey HSD post hoc test. The other diversity indices showed no significant differences between the plants. Previous studies have shown that the alpha diversity of highly symptomatic citrus plants is significantly different from that of asymptomatic plants, and here we aimed to compare mainly the effects of herbicide/insecticide and fertilizer treatment on the diversity of managed citrus plants at a low symptomatic level and asymptomatic non-managed grove sites.

**Figure 7:**
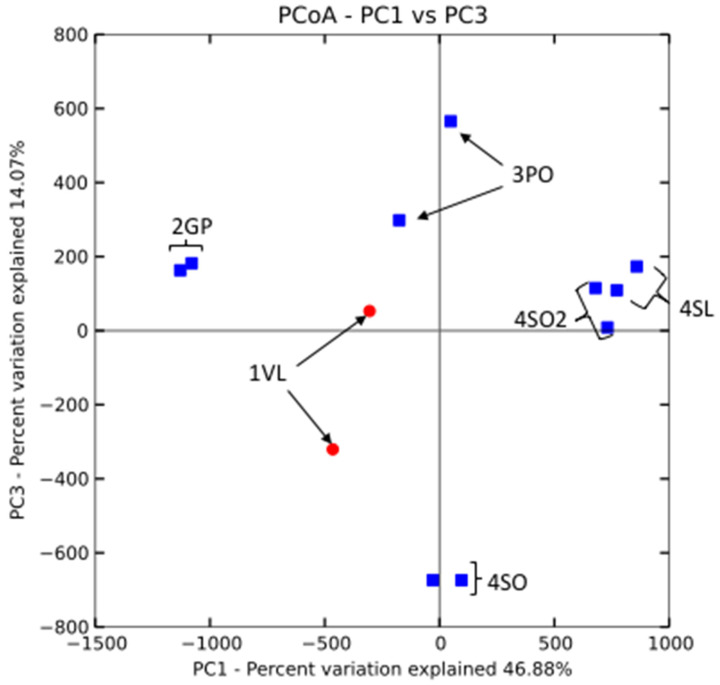
Principal coordinate analysis for the 16S rDNA gene using a non-phylogenetic Euclidean distance matrix of the managed sweet Valencia (1VL—red) and other non-managed Citrus plant types/varieties (blue), showcasing their clustering patterns.

Beta diversity PCoA cluster analysis of all the sequence data is shown in Figure 7 with 46.88% and 14.07% variation represented on the PC1 and PC3 axes, respectively. From the PCoA plot, both Valencia rhizosphere replicates (1VL—red) clustered away from the non-managed citrus plant rhizospheres (blue). The non-managed citrus plant types clustered very close to each other along both axes, where sour orange HCT (4SO2) and sour lemon (4SL) had a similar clustering pattern. Pineapple orange (3PO) clustered away from the sour orange plant (4SO), all from non-managed sites (blue).

## 4. Discussion

The important role played by soil health and root-resident microbes in pathogen suppression of root-borne diseases is well established. Growing evidence equally emphasizes their involvement in systemic plant immunity against foliar and arthropod-borne diseases through a process called induced systemic resistance (ISR) and more. Plants infested with insects or pathogens release exudates, which attract microbes that trigger ISR. The ISR signals are known to travel from the roots to shoots and leaves, activating enzymes, proteins, and volatile organics in defense pathways that fortify plant immunity [24]. This process has not been well studied for the Citrus greening disease, whose complex pathogenesis is in part fueled by the plant’s immune response. Nevertheless, data on bacterial community structure and CGD (Ca L. asiaticus) detection in undisturbed soils presented here (Figure 3, Figure 4 and Figure 5) suggest that some poorly known beneficial microbial consortia, depleted in heavily managed groves, could account for the low to no CGD, at least in part. Although the population of the disease insect vector Diaphorina citri was not measured at the managed and undisturbed sites, the close proximity of our study locations (Figure 1) strongly suggests the ubiquity of the insect in this region.

The plant rhizosphere microbial community is considered one of the most diverse, and many members support the growth of their plant hosts. Many of these microbes are involved in key processes such as nitrogen, phosphorous, and carbon cycling, and help defend plants against soil pathogens through competition and immune response regulation. Distinct shifts in the microbial community structure of the plant rhizosphere will have deleterious effects on the ecosystem function, interfering with nutrient uptake, growth, defense, and overall health. The phytopathogen infection has been shown to cause shifts in plant exudate production [25] and subsequently the microbial community diversity in many infected plants. This phenomenon has been previously shown in many different plants, including Citrus [4,11,26,27]. Using 16S rDNA analysis, this study investigated the bacterial community shifts that occur in Citrus plants at various levels of infection and explored, possibly for the first time, the bacterial community structure and abundance of the rhizobiome under historically non-managed citrus groves in Central Florida using 16S rDNA amplicon sequencing. Although the presence of genomic DNA may equate to the microorganisms that are active in the ecosystem, it has been proven on most occasions that the dominant species identified by mRNA are also the most abundant in genomic DNA [28].

Furthermore, the root exudates of plants differ between varieties and species and could account for differences in 16S amplicon-based community profiles. One of the most intriguing observations in this study is that the microbiome of different types (Lemon, Valencia, Grapefruit) of healthy citrus plants growing in non-managed soils clustered similarly regardless of the site (Figure 3, Figure 4 and Figure 7), implying that major drivers of the bacterial community shifts reported here are external to the plant/microbe interaction: the application of agrochemicals in this case.

The study showed that a drastic change at the phylum level in the bacterial community structure occurred only after a high level of infection was observed in the West Valencia plants at managed site 1, where there were heavy signs of HLB infection. Remarkably, the East Valencia plant at the same site, also infected, but showing milder symptoms, mimicked the bacterial community structure of the non-infected citrus plants on the non-managed sites, where the Proteobacteria phylum was most prevalent and the Firmicutes had low prevalence. There was an increase in the relative abundance of the Firmicutes phylum and a subsequent decrease in the Proteobacteria phylum (Figure 5a), similar to what was previously found [4] for the highly infected West Valencia. There was also a significant decrease in the Shannon’s and Simpson’s diversity indices for the West Valencia plant, while the East Valencia plant at the same site had similar diversity indices to the non-managed uninfected Citrus plants (Figure 6). The West Valencia plants clustered well away from the other plant types, but similar species from the non-managed Citrus plants clustered with each other (Figure 7). They likely clustered differently due to the plants being of different citrus species and varieties, where the exudate and bacteria recruitment profiles differ slightly. There was still some variation in the clustering pattern of the East Valencia replicates, likely due to high variations in the relative abundance of some key taxa, such as *Acidobacterium* sp., *Rhodoplanes* sp., and other members of the *Alphaproteobacteria* class (Table 1). Infection by the HLB pathogen causes phloem restriction in Citrus plants, preventing the allocation of nutrients throughout the plant [1], where the roots are eventually starved of nutrients. Bacterial members with high activity, metabolism, and growth will be the first ones to be eliminated, as observed in the Proteobacteria phylum, where members of the *Alphaproteobacteria* were reduced under the highly infected West Valencia. Specifically, bacteria involved in nitrogen fixation, such as *Bradyrhizobium* sp., *Rhizobiales* order, and *Rhodoplanes* sp. (Table 2), were significantly reduced. There were also other numerous bacterial taxa missing from the rhizosphere of the West Valencia plant but were found in the East Valencia and Citrus plants on the managed sites. When these copiotrophic bacteria are eliminated, the more oligotrophic and slow-growing forms will persist [29]. In addition, according to a study, a general progression of more pathogenic, competitive, and saprophytic bacteria and fungi will eventually fulfill the niche [11]. Evidence to support this was seen in the Firmicutes phylum under the infected West Valencia, where there was a 13-fold increase in its prevalence compared to the other Citrus plants, with Bacillus sp. representing close to 50% of the total bacterial reads and 70% of the Firmicutes reads. Bacillus sp. is known to play many roles in the soil ecosystem, being a dominant member of bulk soil and also aiding in plant protection in the rhizosphere. The Bacillus sp. is very resilient and has been shown to survive fluxes in temperature and pH in the plant rhizosphere [30]. The *Bacillus* sp. is also spore-forming, which could be a probable reason for its increased prevalence in the diseased West Valencia plant.

Citrus species at non-managed historical sites devoid of anthropogenic influence (herbicide/fertilizer treatment) have never been fully explored to determine if a stable core rhizobiome exists with potentially beneficial microbes that could be used to improve plant health and productivity. Herbicide treatment, which in this case involves the use of glyphosate (Roundup^®^), has been shown to have an overall benign effect on the soil microbial community, only eliciting a significant microbial community shift at application rates higher than recommended [31]. It is likely, then, to expect that if recommended application rates were followed on the managed citrus grove site, as reported (Dr. D. McCorquodale, Pers Comm, Dec 2017), the microbial community structure would not be substantially affected. The use of commercial fertilizers, however, can greatly affect the abundance, microbial community structure, and function of soil bacteria [32] at the managed sites, where the number of nitrogen-fixing, phosphate-solubilizing bacteria and fungi may be reduced due to lesser plant dependence on these microbes and niche reduction. Also, it shows that the application of commercial NPK fertilizers reduced members of the phylum *Acidobacteria, Nitrospira, Chloroflexi,* and *Planctomycetes* while enhancing members such as *Sphingomonas* spp. [33]. Thus, it is imperative that the continued application of fertilizers in managed citrus groves may reduce the formation of a stable core microbiome of beneficial bacteria, unlike the more natural non-managed sites. It is imperative to continue investigating the optimal thresholds of agrochemicals to be employed in current agricultural practices for the purpose of lowering induced dysbiosis. These non-managed sites may contain a richer and more stable core microbiome to be further explored. In our findings, the bacterial community structure of the grapefruit, pineapple orange, sour orange, and sour lemon plant rhizospheres was somewhat consistent with one another (Figure 7), where the Proteobacteria phylum was the most prevalent, and the Firmicutes and *Verrucomicrobia* phyla had the lowest prevalence. They also shared approximately the same relative abundance of Firmicutes with the infected East Valencia, which exhibited mild symptoms. Some of these members were described as putative plant-growth-promoting bacteria in the citrus rhizosphere [11,20]. There was also a significant increase in members of the *Verrucomicrobia* phylum in a few non-managed sites. Even though many of these members are still unknown, one study has shown that they are extremely sensitive to changes in soil fertility [33]. The reduction of these and potentially other beneficial organisms could prevent the recovery of these diseased plants under biotic and abiotic stress. A recent study has shown that the inoculation of beneficial plant-associated bacteria, such as *Bacteriodetes* sp., can initiate defense responses in plants to help confer resistance. There is a substantial, stable core microbiome that exists in the non-managed citrus rhizosphere to be explored and exploited to create synthetic microbial communities to restore plant health and productivity. Similar studies will have future implications and benefits in improving the sustainability of agricultural and non-agricultural crops as we begin to explore the core microbiome, and the major players are identified in healthy, anthropogenic-free plant crops.

## 5. Conclusions

Agricultural management practices, such as the copious use of agrochemicals and fertilizers, impact plant microbiomes via direct inhibition of plant pathogen inhibitors and indirect feedback suppression of beneficial bacteria. In addition, plants infected by phytopathogens can cause drastic shifts in the microbial community structure, affecting the overall health of the plant. We have shown here that the detrimental changes to the microbial community structure are subtle and are not readily captured by diversity indices and high taxonomic order measurements. A detailed resolution of the microbial functional groups and lower taxa analysis is more revealing.

This unique report on the core microbiome of non-managed 130-year-old citrus groves and cultivated sites in the same vicinity in Central Florida provides some evidence that the massive use of antimicrobial herbicides, pesticides, and fertilizers could alter key beneficial members of the core microbiota of citrus involved in plant immunity to CGD. We have shed some light on the different taxa (many unknown) that are maintained in the rhizosphere without anthropogenic influences. These taxa could be the key to unlocking potentially beneficial bacteria that may have been depleted in degraded soils through consistent chemical herbicide and fertilizer use on managed groves. Restoration effects and sustainability interventions aimed at improving soil health and plant resilience should include microbiome-based solutions using synthetic consortia or via bio-stimulation.

## Figures and Tables

**Table 1:** Prevalence of *Liberibacter asiaticus*—among various citrus plants in cultivated groves and non-managed undisturbed sites (qPCR detection).

Plant Types	Site	Collection Date	qPCR Results	Sample Location History	Additional Remarks
Valencia	1	25 November 2016	POS	Grove Hendry Co. FL. 26.77° N, 81.18° W	Cultivated 30+ years—herbicide and fertilizer treatment
Valencia	1	25 November 2016	POS	Grove Hendry Co. 26.77° N, 81.18° W	Cultivated 30+ years—herbicide and fertilizer treatment
Natural Stand Grapefruit	2	25 November 2016	NEG	Hammock Hendry Co. FL 26.61° N, 81.13° W	Planted 1880s No use of chemicals Isolated area
Greenmount Pineapple Orange	3	18 February 2017	NEG	Pasture Arcadia FL 27.21° N, 81.83° W	Isolated area
Hart Highway Sour Orange	4	17 February 2017	NEG	Sebring FL 27.42° N, 81.56° W	Non-grove Wild with seeds
Hart Cracker Trail Sour Orange	4	18 February 2017	NEG	Highland Hammock State Park 27.42° N, 81.56° W	Non-grove Wild with seeds
Hart Cracker Trail Sour Lemon w/roots	4	17 February 2017	NEG	FL Cracker Trail 27.42° N, 81.56° W	Non-grove Wild with seeds

**Table 2:** One-way ANOVA analysis of the relative abundance of key bacterial taxa under the rhizospheres of each Citrus plant type. *p*-values represent any significant differences in the relative abundance under the Valencia rhizosphere (managed) compared with the other Citrus plants (non-managed), confirmed by the Tukey HSD post hoc test, numbers with same letter are not significantly different.

	Managed	Non-Managed					
Bacteria taxa	1VL	2GP	3PO	4SL	4SO	4SO2	*p*-value
***Acidobacterium*** **sp.**	1.28 ± 0.34 ^a^	0.71 ± 0.03 ^ab^	0.15 ± 0.003 ^b^	0.20 ± 0.03 ^b^	0.30 ± 0.1 ^b^	0.13 ± 0,01 ^b^	**0.001**
**Actinomycetales order**	0.61 ± 0.09	0.55 ± 0.25	1.02 ± 0.33	2.20 ± 1.50	2.43 ± 1.30	1.87 ± 1.12	0.33
**Micrococcales order**	0.23 ± 0.03 ^a^	0.02 ± 0.006 ^b^	0.04 ± 0.004 ^b^	0.02 ± 0.01 ^b^	0.05 ± 0.02 ^b^	0.02 ± 0.01 ^b^	**<0.001**
**Actinobacteria class**	2.00 ± 0.03	2.12 ± 0.06	4.10 ± 0.25	4.31 ± 1.06	5.33 ± 1.74	4.02 ± 1.44	0.089
***Nitrospira*** **sp.**	1.50 ± 1.21 ^a^	0.94 ± 0.22 ^b^	0.92 ± 0.13 ^b^	0.34 ± 0.02 ^b^	1.14 ± 0.09 ^ab^	0.23 ± 0.05 ^c^	**<0.001**
***Bradyrhizobium*** **sp.**	1.09 ± 0.32	2.05 ± 0.47	2.68 ± 0.55	3.40 ± 0.88	1.77 ± 0.85	2.79 ± 1.79	0.109
***Rhodoplanes*** **sp.**	0.73 ± 0.15 ^a^	0.30 ± 0.08 ^ab^	0.16 ± 0.04 ^b^	0.20 ± 0.05 ^b^	0.14 ± 0.02 ^b^	0.21 ± 0.02 ^b^	**0.002**
**Rhodospirallaceae family**	1.43 ± 0.78 ^a^	0.18 ± 0.04 ^ab^	0.19 ± 0.06 ^ab^	0.06 ± 0.01 ^b^	0.11 ± 0.01 ^b^	0.05 ± 0.02 ^b^	**0.0028**
***Sphingomonas*** **sp.**	1.94 ± 0.11 ^a^	1.59 ± 0 ^ab^	0.99 ± 0.17 ^bd^	0.38 ± 0.13 ^cd^	0.87 ± 0.14 ^bd^	0.94 ± 0.42 ^bd^	**0.003**
**Betaproteopacteria class**	0.81 ± 0.09 ^a^	0.25 ± 0.06 ^ac^	1.03 ± 0.36 ^ab^	2.04 ± 0.49 ^b^	2.67 ± 0.11 ^b^	2.15 ± 0.01 ^b^	**<0.001**
**Xanthomonadales class**	3.07 ± 0.17 ^a^	7.88 ±1.19 ^b^	0.03 ± 0 ^c^	0.32 ± 0.14 ^c^	0.18 ± 0.02 ^c^	0.24 ± 0.07 ^c^	**<0.001**
**Verrucomicrobia phylum**	0.84 ± 0.06 ^ab^	0.45 ± 0.02 ^a^	1.44 ± 0.32 ^ab^	2.34 ± 0.68 ^b^	4.68 ± 0.56 ^c^	2.34 ± 0.06 ^b^	**<0.001**

## Data Availability

The original 16S sequence files were submitted to NCBI with project accession #PRJNA680160 and sample accession numbers SRX9584730-43.
